# Going against the Grain: Overturning the received wisdom of a field takes not just strong evidence, but a strong personality. For one reproductive biologist, only time will tell whether such challenges are worth the risks in today's pressurized research climate.

**DOI:** 10.1371/journal.pbio.0050338

**Published:** 2007-12-27

**Authors:** Kendall Powell

## Abstract

A reproductive biologist's research at Massachusetts General Hospital in Boston has stoked a controversy about whether female mammals can renew egg cells.

The data that would change the course of Jonathan Tilly's career and cause an uproar in the field of ovarian biology almost never saw the light of day. In Tilly's cell death lab, postdoctoral fellow Tomoko Kaneko had twice repeated her experiments to kill off mouse egg cells, but something was wrong because the egg cell numbers were still high after treatment with a chemotherapy drug. Kaneko consulted another postdoc in the lab, Josh Johnson, and together they tried to determine if she had made a technical mistake or perhaps switched her control and experimental groups.

“All of us ‘knew’ that egg regeneration couldn't be occurring,” says Johnson, referring to the long-held view that adult female mammals are born with a fixed pool of oocytes, or egg cells, which gradually declines in number with age. The work appeared to be an anomaly, but Johnson prodded Kaneko to take it to their advisor's office. That 2002 meeting was the birth of an ongoing controversy that has shaken up the field of reproductive biology, with Tilly's laboratory publishing data they interpret as evidence of egg regeneration occurring in adult mice.

**“All of us ‘knew’ that egg regeneration couldn't be occurring.”**

Convinced that the dogma of a fixed pool of oocytes is wrong, Tilly, a reproductive biologist at Massachusetts General Hospital in Boston, has brought this challenge forth almost single-handedly. His first paper, published in *Nature* in 2004 [1], and the exposure it received in the mainstream media, brought on a slew of harsh criticism and skepticism from senior researchers in the field of ovarian biology. In the years since, Tilly's group has published two more papers [2,3] extending the story, and his critics have published a handful of papers refuting his claims [4,5]. In addition, independent groups have published findings that both sides claim support their views.

For most researchers familiar with the controversy, the matter is not resolved and continues to stimulate discussion and new work. But the debate has become highly polarized and somewhat adversarial, with most players falling into two camps—those who think Tilly's challenge and the idea of regeneration hold merit and those who hold firm to the dogma and dismiss Tilly as misguided. The stakes for this debate are high—practically all research done in the field of ovarian biology in the past 100 years has been grounded in the fixed-pool dogma. More importantly, perhaps, this idea has also shaped the way in which doctors treat women for infertility and menopause. If the dogma were overturned, it would mean that current treatments for female reproductive problems—for example, infertility treatments after cancer therapy or for aging women, and for ovarian failure, a condition related to menopause—may be based on false assumptions.

“Controversy is a very vital part of the scientific process,” says Roger Gosden, an ovarian expert at Weill Cornell Medical School in New York City. “It's the critical nature of science which gives it its strength and authority.” Though critical of Tilly's interpretations, Gosden admits that the question of regeneration is so important to the field of reproductive biology that it should be investigated thoroughly.

This particular controversy makes a compelling case study for exploring how challenges to scientific dogma proceed in today's research climate. It carries historical perspective, because the field experienced the same challenge roughly 75 years ago. It also raises questions about challenging ideas in the information age, when new research breakthroughs are just a click away from patients' fingertips. Tilly's confident, bold style raises the issue of whether a less pushy approach would meet less resistance.

This controversy also highlights the problems with replicating studies to confirm or refute findings. Whose burden is it to do these experiments, and under current pressures, how best to get that work done? What standard of evidence is needed to overturn an entrenched idea?

## Reigniting a Past Debate

In 1870, German anatomist Heinrich Wilhelm Gottfried von Waldeyer-Hartz first proposed that oogenesis, the making of new oocytes, occurs before birth in female mammals and results in a finite number of oocytes that will decline during a lifespan. This view had been challenged by two researchers in the early 1920s and 1930s, when Lord Solomon “Solly”

Zuckerman, then a young professor at University of Birmingham in England, undertook a series of experiments to investigate the challenge. He began his quest convinced the dogma was wrong and was inspired by the thought that “one ought to be able to promote oogenesis in a failing ovary...of a menopausal woman. This notion was more than enough bait to stimulate further enquiry.” [6].

Zuckerman then proceeded to spend two decades exploring the challenges to the dogma by using the new histological techniques and knowledge about the cyclical nature of the female sexual hormones. In 1950, he gave a seminal talk at the Laurentian Hormone Conference of the American Association for the Advancement of Science (AAAS) in Franconia, New Hampshire [7], in which he summarized his body of work before the leading reproductive biologists, many of whom were anticipating he would provide evidence of oogenesis in adult life. Instead, Zuckerman produced a mountain of evidence that reinforced the dogma. The chair of the session concluded with the remark “Waldeyer must have been right after all,” and the audience filed out with a feeling that the question was resolved.

Both Waldeyer and Zuckerman's work, the bricks of the dogma's foundation, rests largely on the simple process of counting egg cells—or more specifically, the follicle structures that encase egg cells inside the ovary—over the course of an animal's lifetime.

**If the dogma were overturned, it would mean that current treatments for female reproductive problems … may be based on false assumptions**.

**Figure 1:**
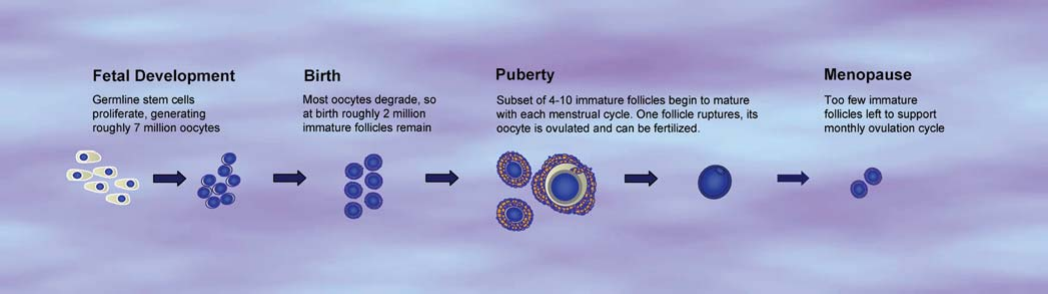
Oocyte Development During the fifth month of fetal development in female humans, roughly 7 million oocytes are produced within the ovaries from germline stem cells. These primary oocytes will be surrounded by flattened cells to form primordial follicles by the seventh month. At birth, many of these oocytes will have degenerated, leaving roughly 2 million oocytes. Follicle development then ceases until puberty, when follicles mature during each ovulation cycle. Only a fraction of these oocytes will be ovulated and potentially fertilized between puberty and menopause, roughly 400–500 cells if a woman ovulates regularly each month. The rest of the oocytes remain in the ovaries as immature oocytes. The two types of follicles—immature follicles and those destined to be ovulated—play different roles during the menstrual cycle. Some of the immature follicles produce signals that, along with hormones secreted by the brain, support the development of a subset of 4–10 primary follicles, which grow in size. Eventually, one follicle becomes dominant, grows larger, ruptures through the ovary wall, and releases its egg cell into the reproductive tract. After ovulation, a wave of cell death, or atresia, destroys some of the follicles that were growing, but not ovulated. Over a lifetime, this monthly cycle of ovulation and cell death depletes the population of immature oocytes to the point where they can no longer support ovulation, and menopause ensues.

Such follicle counting would seem relatively straightforward. But how to properly count these tiny structures—which grow, change form, and die off over a monthly cycle inside a rather inaccessible and complex organ—has been the subject of much debate from the 1880s onward (for a primer on egg development, see Figure 1). Add to that the logistical and ethical impossibilities involved in investigating oocyte numbers from healthy human ovarian tissue, and simple counting starts to seem rather vexing.

The current controversy (Box 1) has some striking differences from that of Zuckerman's time. Seventy-five years ago, the debate took place almost exclusively within the halls of academia and in the literature—removed from the public consciousness. Infertility treatment did not yet exist. Zuckerman and his peers argued over the scientific evidence but did not touch upon implications for patient care or patient concerns.

**Box 1. The Making of a Controversy**

The first paper Tilly, published in *Nature* in March 2004, was based on the fact that follicle numbers did not add up in the mouse chemotherapy experiments that his postdoc Josh Johnson repeated [1]. The authors postulated that an ovarian stem cell must be regenerating oocytes. In the next paper, published in *Cell* in July 2005 [2], the group reported that stem cells from the bone marrow replenish the egg supply in the chemotherapy-treated mice.

In 2006, two reports were published from other groups, one of which appeared to support Tilly's hypothesis and one which appeared to refute it. Roger Gosden and Amy Wagers published a study in *Nature* that was designed to test whether such putative stem cells contributed to ovulated eggs [4]. Their experiments showed that mice sharing a circulatory system, and presumably any such bone marrow-derived stem cells, only ovulated their own eggs. At this point, many scientists in the field felt the issue had been laid to rest since, in their view, the ultimate physiological significance of egg regeneration would be to contribute new ovulated eggs and offspring. But Tilly and other independent scientists pointed out several technical flaws with the Wagers study. Tilly's biggest complaint was that the study did not include an analysis of what was happening inside the ovaries of these animals—to show the presence or absence of donor-derived stem cells or oocytes—but only looked at ovulated eggs.

The other paper published in 2006 bolstered Tilly's work. Jeff Kerr and Jock Findlay, reproductive biologists from Monash University and Prince Henry's Institute of Medical Research, respectively, in Clayton, Australia, produced a quantitative study that tracked healthy follicles in mice through adult life [10]. Although they found no evidence for germline stem cells or another mechanism to explain their results, the team's data supported the hypothesis that follicle renewal was occurring in the adult mice.

Tilly sees this study as a replication of sorts of the follicle numbers finding from his first paper. Others in the field, although hard-pressed to find technical fault with Kerr and Findlay's experiments, say the results are open to different interpretations based on how the statistical analysis was performed.

**Figure:**
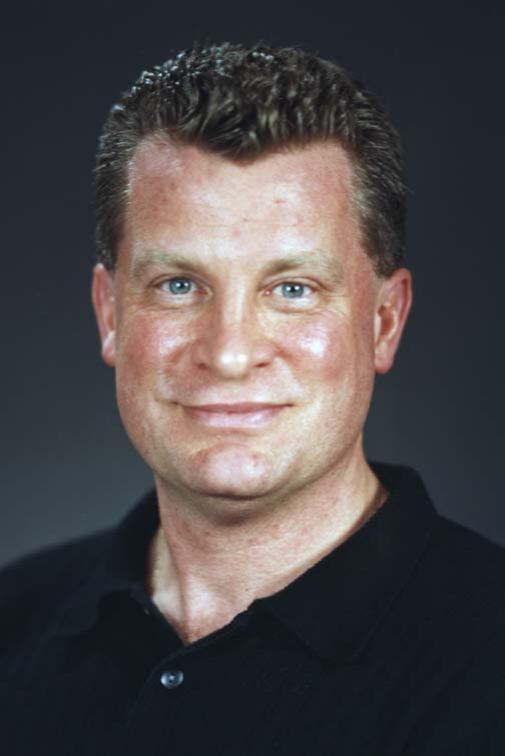
**Jonathan Tilly's research at Massachusetts General Hospital in Boston has stoked a controversy about whether female mammals can renew egg cells**. (Image credit: Massachusetts General Hospital public affairs)

“It makes a huge difference if a controversy is conducted in private or in public,” says John Durant, who directs the MIT museum in Cambridge, Massachusetts, and studies the relationship between science and the public. With medically relevant science, he says, there is the potential for the public to seize on findings that could be premature at best and false at worst.

“Once you've interested the public in what you are doing, you cannot shut the door,” says Durant. Indeed, many of Tilly's critics are most upset that his first paper's findings were splashed across the headlines of major newspapers and that Tilly was freely discussing the potential human health implications in the media.

The current trend in fertility treatment—which focuses on preserving fertility in both cancer patients and aging women—adds to the consternation of Tilly's critics. David Albertini, an oocyte expert at Kansas University Medical Center in Kansas City, reports meeting an increasing number of women patients at conferences on fertility preservation who have educated themselves on techniques not yet available in fertility clinics.

Albertini, one of Tilly's most vocal critics, says it would have been healthier for the field if Tilly's first announcement had been more “subtle” both in the literature and in the media. “Phones were ringing off the hook with patients calling clinics long before the scientific community had a chance to evaluate that work,” he says.

Tilly feels his comments to the press were an appropriate balance of optimism and caution, and defends his stance: “Yes, I tell people what I think this work may achieve for humans. How can you get criticized for speculating about the meaning of your work?”

In contrast to arguments occurring behind the ivory tower walls, Durant says publicized debates become less predictable and more polarized. Evelyn Telfer, an oocyte researcher at University of Edinburgh, UK, points out another key difference facing researchers today: “Universities are operating within a market environment now, and there is more pressure to get your results into the public domain and get patents,” she says.

## Risking Reputation or Pursuing a Vision?

A good portion of the skepticism directed at Tilly's work stems not so much from the scientific arguments, but from the very fact that only one group is challenging the dogma. Many scientists are reluctant to even weigh in officially on the debate until other groups come forward with similar, supporting work.

“What anyone publishes is not really the corpus of scientific knowledge unless it can be verified,” says Gosden. “You don't get a paradigm change until you have a consensus of expert opinion,” he says, and that is certainly not the case here. This follows physicist and science historian Thomas Kuhn's view of scientific revolutions—that many inconsistencies must build up in a field of science before a paradigm shift can occur.

The Kuhn model of paradigm shifts describes how most central ideas in science get revised or overturned, but there is another model that some would argue is equally valid, if rarer: the “lone voice in the wilderness” of a single scientist pushing a revolutionary idea forward. There are notable examples of lone voices who both succeeded and failed in overturning an idea. Stanley Prusiner's hypothesis that aberrant protein structures called prions could cause infection initially lost him his bid for tenure at the University of California at San Francisco and significant funding from the Howard Hughes Medical Institute, but eventually won him a Nobel prize in physiology or medicine in 1997. The cold fusion work of the late 1980s, which was never replicated to the satisfaction of the nuclear physics community, eventually resulted in Martin Fleischmann and Stanley Pons dropping out of academia.

“There's a fine line between being a maverick and a genius prescient person,” says Durant. Currently, Tilly is gingerly straddling that line, which can mean the difference between receiving the highest scientific honors or the scorn of your peers.

“Jonathan is very good at putting his points across and defending his work very forcibly, and he has a right to do so,” says Telfer. “But he can upset some people because he's presented himself as a misunderstood visionary and that everyone else just doesn't ‘get it.’” Telfer led the publishing of one of the most scathing commentaries on Tilly's first paper [8], but has since softened her stance toward his work, noting that he has addressed well some of the scientific criticisms of his work in follow-up studies.

Tilly knows what he's risking in pursuing his challenge to the dogma, but says he's decided to commit his laboratory to this line of work, because the issue remains unresolved, the answer is too important, and he has too much data to believe he is wrong. It has changed the type of junior scientists who apply to his group, weeding out those who don't want to get involved in the controversy, but that isn't all bad in his view.

“This isn't something that someone weak of conviction or heart would want to take on,” he says. “You have to be confident about your abilities to do this work and remain fairly insulated from criticism.”

And some argue that science can benefit from big personalities. “Whether concepts in biology and medicine evolve has a lot to do with whether or not there are strong-voiced, strong-willed champions,” says David Scadden, director of the Harvard Stem Cell Institute and a co-author on Tilly's bone marrow study. Tilly claims he didn't seek out this controversy, that he stumbled into it from his group's work in cell death, but it's clear he enjoys the limelight to a certain extent. It's also clear that circumstances of his position offered him some protection to become the squeaky wheel. His group had already distinguished itself with a decade of solid research in the apoptosis field, and he'd been promoted from assistant to associate professor within Harvard Medical School. His institution enjoys a large amount of philanthropic support, making its researchers somewhat less dependent on federal grant money.

Such security stands Tilly in stark contrast to his former postdoc, Johnson, who moved to Yale University School of Medicine as an assistant professor in 2005. Johnson's lead authorship on the two high-profile publications from his postdoc project helped his search for a faculty position, but funding for extending his postdoctoral work has been extremely difficult to come by.

“There is a skeptical undercurrent in the field that occasionally comes to the surface,” Johnson says. “I have received advice at meetings from senior researchers in the ovarian field to distance myself from Jonathan and my postdoctoral work in general, at least for now.” As such, Johnson has broadened his interests and research program to the general effects of stress on oocyte quality, ovarian function, and fertility. He admits he's a little jealous of colleagues whose transitions from postdoc to professor were smoother than what he's experienced.

“But I unreservedly believe in the data we got.” He also says he would volunteer for the project if he were back in Tilly's office again. “The data is there, the question is there, and you have to follow it up,” he says. “It's not about the hype or the politics or the hurt feelings—honestly it's the answer that matters: how does the ovary really work?”

Tilly does worry that the controversy will affect his next tenure promotion and that it may impede the publication record of his fellows. And he hopes he won't flee academia like other lone voices who turned out to be wrong. But, he says it's not in his personality to back down from a scientific puzzle. “It's my responsibility as a scientist to go after it and figure it out.”

It took Prusiner decades to fully convince his critics about prions and change a field's view of infectious mechanisms. Sometimes Tilly feels as if his critics want “20 years of work in four years”.

The latest piece of the puzzle, published in August 2007, showed that mice receiving bone marrow transplants after chemotherapy treatment recover fertility and that donor-derived cells appear as immature follicle structures in the recipient mouse's ovary [3]. Although none of the treated mice gave birth to pups arising from donor-derived eggs (all pups were genetically matched to the mother), Tilly interprets the appearance of the donor-derived immature oocytes as evidence for regeneration of immature “helper egg follicles,” which restore fertility in what would otherwise be sterile animals.

Also this year, Albertini and his colleague David Keefe of the University of South Florida in Tampa published a study analyzing adult human ovarian tissue for genetic markers of meiosis and germ cell proliferation, both of which would be expected if a germline stem cell were present and regenerating oocytes in adult human ovaries [5]. They found no evidence of such markers.

Albertini now characterizes the debate as lingering among physicians, but among basic researchers as a closed case against women being able to regenerate oocytes. Many others, including Telfer and Gosden, say the door is still open for more work to be done and that the “helper follicle” idea may very well hold water. But no matter which way the dice eventually roll for Tilly's ideas, the debate itself has already changed the field.

## Harmful or Healthy?

Telfer goes so far as to say that Tilly “has done a service to the ovarian biology community.” She says his work has stimulated new approaches to her laboratory's studies on oocyte maturation. And she credits the debate for spawning new questions about the “helper follicle” hypothesis, the timing of follicle formation, and whether oocytes are flexible to manipulation, even if they are in a fixed pool.

Gosden agrees the controversy has cast a spotlight on new questions that would not have been investigated otherwise. And he doesn't see any harm in revisiting the dogma with new technologies, quoting T.S. Eliot's poem Little Gidding, “At the end of all our exploring/Will be to arrive where we started/And know the place for the first time.”

Amy Wagers, a stem cell biologist at Harvard's Joslin Diabetes Center in Boston, points to other controversies in which the original interpretation proved wrong but led to the discovery of a novel process, nonetheless. For example, in stem cell biology, claims of transdifferentiation—one cell type switching fates to become another cell type—eventually gave way to the finding that cells were fusing to become functional hybrid cells. “We now know that cells can do something we didn't know before, and it raises important questions about whether cell fusion is important in normal physiology or in disease,” says Wagers.

But Albertini says the controversy has been “devastating to the field as a specialty area” because it hasn't been formally resolved. He and others such as Keefe feel strongly that the medical community has been misled into believing there are implications for human fertility preservation. When future therapies fail to appear, it will tarnish the image of reproductive biologists. Albertini also sees the hunt for ovarian germline stem cells as a distraction that has diverted substantial time and resources to repeating work that he contends was not up to par in the first place (Box 2).

**Box 2. Wrestling with Replication**

The current climate of scientific debate has been changed by modern influences on public communication and by funding and publication pressures, which have also complicated the process of replicating results.

After Tilly's first two papers were published, the implications spurred high interest in the field for finding a way to replicate or refute his findings—but exactly repeating his group's mouse experiments could easily take another group five years or more. Instead, Gosden decided to appeal to several hematology groups, including Wagers', to find a collaborator who might have the “spare” animals and resources to test more quickly the idea that stem cells from bone marrow were responsible for regenerated oocytes.

“Replication is a huge problem—all of that work is not funded and not fundable, especially in today's climate,” Wagers says.

Exact repetition of experiments will not fly, Wagers adds, unless you have somehow extended the original study. “But if you have discovered something fundamental, it should be arrived at in multiple ways by multiple groups—that is the mark of an important discovery.”

Tilly, however, does not count the Gosden and Wagers paper as a valid attempt at replicating his work—mainly because it did not do the same types of experiments his group did and because the group used a different strain of green fluorescent protein (GFP)-marked mice. (In Tilly's strain, only the germline cells are fluorescent green and in the Wagers' mouse line, every cell expresses the green dye.)

But his biggest gripe with the study brings up the issue of who should shoulder the burden of verifying or refuting claims made by another group. In the Wagers paper, the authors noted: “Although cells derived from circulation may be found within the ovaries of parabiotic animals, these engrafted cells exhibit exclusively haematopoietic fates, and probably represent circulating blood cells known to infiltrate all tissues...as immune responders.” [4].

Tilly's critics seized on this sentence as “proof” that the bone marrow–derived cells he was seeing in mice ovaries were not regenerated oocytes, but rather, immune cells activated to offset the chemotherapy-induced damage. At the time the Wagers paper was published, Tilly was incensed that such a statement could appear in a *Nature* paper without supporting data, such as immune cell markers, to back it up.

“The burden isn't on us to go back and prove their claim is true,” he said in a 2006 interview. But eventually his group did end up addressing the issue experimentally, because the “immune cell” criticism persisted. His group's latest publication includes an experiment showing that the GFP-marked cells from their mice, some of which end up in the bone marrow transplant recipient's ovaries, do not express immune cell markers [3].

Tilly acknowledges that a direct repeat of his group's body of work is too high a bar to set, but he says individual experiments could easily and inexpensively be repeated. For example, an experiment from the 2005 *Cell* paper [2], which treats mice with a chemotherapy drug and then counts follicles afterward, could be done in two weeks for a few hundred dollars, he says. Why no one has bothered to do it yet, he says, is more telling about his critics' motives than a lack of resources.

“They don't want to do it because they might find something they don't want to find,” says Tilly. “If someone tried to do this experiment and could not repeat our results, for this particular topic, that would be very publishable in a high-profile journal.”

Troubles with replicating work, which have especially plagued the stem cell field, often cast doubts on certain groups but also occur more commonly than scientists like to admit [11]. “The ideal is that we will do this experiment and get the predicted result or not, which will confirm or falsify our hypothesis,” says Durant of the MIT museum. “But in practice, the attempts are not so straightforward. The question of what counts as a critical test and any given outcome of the test are open to debate.”

**“It's not about the hype or the politics or the hurt feelings—honestly it's the answer that matters: how does the ovary *really* work?”**

Although these arguments play out in the literature and conference presentations, both camps have alternately charged that certain experiments from the other side could not be repeated or that data have been suppressed. Though some fear that such accusations are damaging the field, MIT's Durant counters that it's a normal part of scientific discourse and controversy.

“Scientists are very much like everyone else when caught up in a controversy and will often reach for any and all resources to support their position and undermine their opponent's position,” he says. In fact, it's been shown that there is an asymmetry that exists between the type of rhetoric scientists use to support their own point of view and the rhetoric used to describe their opponent's point of view [9]. Or, as Durant sums up, “*I* have reasons, but *you* have influences.”

Telfer says that the presentation of the debate has been too adversarial and has been cast in absolute terms. “The debate has set up just to be regeneration or not, and that's a shame because that's not really how science should work,” she says, noting that interesting results have been obscured by the shouting over regeneration.

Durant notes that this controversy may not ever come to a clear end like the defining moment of Zuckerman's 1950 talk. “Sometimes the world just moves on, the actors lose interest, and the fickle media turn to another hot topic,” he says. “Science is a lot more like the rest of life—it's messier and more ambiguous than cartoons of science portray.”
